# Overcoming the Cutaneous Barrier with Microemulsions

**DOI:** 10.3390/pharmaceutics6010052

**Published:** 2014-02-28

**Authors:** Luciana B. Lopes

**Affiliations:** 1 Institute of Biomedical Science, University of São Paulo, São Paulo 05508, SP, Brazil; E-Mail: lublopes@usp.br; Tel.: +55-11-3091-7232; 2 Albany College of Pharmacy and Health Sciences, Albany, NY 12208, USA

**Keywords:** skin, microemulsion, transdermal delivery, cutaneous delivery, internal structure, composition

## Abstract

Microemulsions are fluid and isotropic formulations that have been widely studied as delivery systems for a variety of routes, including the skin. In spite of what the name suggests, microemulsions are nanocarriers, and their use as topical delivery systems derives from their multiple advantages compared to other dermatological formulations, such as ease of preparation, thermodynamic stability and penetration-enhancing properties. Composition, charge and internal structure have been reported as determinant factors for the modulation of drug release and cutaneous and transdermal transport. This manuscript aims at reviewing how these and other characteristics affect delivery and make microemulsions appealing for topical and transdermal administration, as well as how they can be modulated during the formulation design to improve the potential and efficacy of the final system.

## 1. Introduction

The skin is a very complex organ, responsible for primary defense, regulation of temperature and sensorial function [1,2]. It is a unique and complex interface and an efficient barrier, designed to minimize the entrance of foreign substances and UV radiation into the body and to prevent the exit of water [1]. However, it not only prevents the entrance of noxious compounds, but also of deliberately applied substances, such as drugs and other compounds of pharmaceutical interest. The barrier function of the skin has been attributed mostly to the organization and composition of the skin’s outermost layer, the stratum corneum, even though a growing number of studies now recognize that skin layers below can also play important roles in the penetration and distribution of drugs [3,4,5].

The skin is used as a route to deliver active agents that are supposed to act in the tissue (cutaneous delivery) and agents that must cross the tissue and be absorbed in the systemic circulation to reach its site of action (transdermal delivery) [6]. The advantages of the cutaneous delivery are obvious, as they relate mostly to localizing the drug directly at its site of action [7]. The advantages of the transdermal route are also well known and include prolonged release and avoidance of first-pass metabolism [3]. Considering the various advantages of the topical and transdermal routes, overcoming the tissue’s barrier is necessary to ensure an efficient delivery of drugs and other active compounds. Various strategies have been developed and employed for this aim, and among them, microemulsions have been extensively studied [8,9,10]. In this manuscript, the use of microemulsions as topical and transdermal delivery systems will be discussed. Due to the broadness of the subject and the extensive amount of material published, we will mainly focus on the influence of the choice and ratio of components and of selected microemulsions characteristics (internal structure, viscosity and charge) on their penetration-enhancing properties. Additional relevant aspects of microemulsions (not only as topical and transdermal delivery systems) can be found in other reviews and books [8,9,11,12,13,14,15,16].

## 2. Basic Concepts

The concept of a microemulsion was first introduced in the 1940s to describe transparent single-phase systems generated by titrating a milky emulsion with hexanol [17]. Since their first description, microemulsions have been extensively studied as delivery systems, due to their multiple advantages. 

In brief, microemulsions are clear, optically isotropic and thermodynamically stable systems generally composed of a blend of oil, water and surfactant(s) [8]. Microemulsions and emulsions are different systems. Microemulsions are clear or translucent and present a small droplet size (generally up to 150 nm) [12,15,18], whereas emulsions are milky, coarse dispersions with droplet sizes generally in the micrometer range and slightly below. Another difference relates to their stability: although emulsions exhibit kinetic stability, they are not thermodynamically stable and will eventually phase-separate [8]. Microemulsions, on the other hand, are thermodynamically stable and form spontaneously (or with very low energy input) under the right conditions [8]. The thermodynamic stability comes with a price: microemulsions require a greater amount of surfactant compared to emulsions, which may result in increased irritation potential. 

The confusion regarding the differences between microemulsions and emulsions was further aggravated by the introduction of the term “nanoemulsion”. Nanoemulsions and microemulsions are described similarly: they are both low-viscosity dispersions with an internal droplet size smaller than 200–250 nm (Table 1) [19]. However, nanoemulsions are kinetically, not thermodynamically, stable. Nanoemulsions present the advantage of being formed with smaller amounts of surfactants, but the preparation of stable nanoemulsions generally requires expensive, high-energy input methods [19]. Another confusion relates to the size of the dispersed phase. Since “micro-” means 10^−6^, while “nano-” refers to 10^−9^, this would imply that the dispersed phase of nanoemulsions is smaller than that of microemulsions. In practice, the opposite is usually true [19]. These differences are summarized in Table 1.

**Table 1:** Comparison of microemulsions with emulsions and nanoemulsions.

Parameter	Microemulsion	Emulsion	Nanoemulsion
Type of dispersion	colloidal	coarse	colloidal
Internal phase size (μm) *	up to 0.15	above 0.5	up to 0.25
Thermodynamic stability	stable	not stable	not stable
Formation	spontaneous	require energy	require energy
Composition	requires greater amounts of surfactant and co-surfactant combination	requires less surfactant	requires less surfactant
**Visual characteristics**			
Consistency	fluid	fluid/semi-solid	fluid
Turbidity	transparent	milky	may vary

* There is no specific value of particle size that represents a definitive cut-off point to differentiate these formulations. The values cited here serve only as guidelines based on papers published on this topic.

As thermodynamically stable systems, the free energy of the colloidal dispersion is lower than the free energy of the separate phases (oil and water) [8,19]. In principle, microemulsions form spontaneously, without energy input. In practice, some energy input (in the form of gentle mixing, stirring or heating) facilitates microemulsion formation, because there are kinetic energy barriers that must be overcome or mass transport limitations retarding formation [19]. Various approaches have been used to explain microemulsion formation and stability [8]; several more in-depth reviews and books are available on this topic, and the reader is encouraged to refer to them [19,20,21,22]. 

In general terms, different types of microemulsions exist, depending on the composition, ratio among compounds and arrangements of the molecules of the components present: oil-in-water (O/W), in which oil droplets are dispersed in water; water-in-oil (W/O), in which water droplets are dispersed in oil; and bicontinuous systems, in which aqueous and oil phases are intertwined and stabilized by sheet-like surfactant regions in the boundary zones between the phases [9,23]. The structure of bicontinuous microemulsions was suggested to have parallels with lyotropic liquid crystals at first; now, it is generally agreed that the structure is not characterized by the same long-range order as liquid crystals, but is rather disorganized or melted [16]. As a consequence of the structure, both the water and oil components are, in principle, free to diffuse over macroscopic distances, and thus, self-diffusion coefficients are high [22]. Multiple types of bicontinuous systems may exist, and other types of aggregates may form between the three main types of microemulsions; appropriate analytical methods are required to accurately differentiate them and identify the structures formed [22]. It is important to note that microemulsions are highly dynamic systems and, as such, undergo continuous and spontaneous fluctuations that consist of phase inversion and changes in droplet size [18]. 

Microemulsions may exhibit percolation phenomena at certain volume fractions of water [11]. According to the percolation theory, phase transformation from reverse structures (W/O) to normal-type systems (O/W) through the emergence of bicontinuous systems and other aggregates may occur as aqueous content in the system increases [10,23]. This is generally accompanied by an increase in the system electrical conductivity, which often has been used as a method for internal structure characterization. Below a critical water volume fraction (Φc), water droplets are embedded in a low conductivity oil medium and isolated from each other. As the volume fraction of water reaches Φc (at the percolation threshold), an increase in the aqueous droplets’ interlinking process and the formation of other structures occur [24,25], which is, most of the times, reflected in a sharp increase in conductivity [10]. Transition into a water-continuous system then follows as the water fraction continues to increase. 

## 3. Why Use Microemulsions as Topical/Transdermal Delivery Systems?

The interest in microemulsions as topical/transdermal delivery systems results from the multiple advantages that these systems present, as described below [8,9,26,27]. Some of the features described here are not exclusive of microemulsions and are displayed by other dermatological formulations and delivery systems. However, few of them combine all the features described as microemulsions do, which provides a reasonable explanation for the popularity of microemulsion use for topical and transdermal delivery. The advantages of microemulsions include the following:

- thermodynamic stability;
- the ease of preparation, as only low energy input is required;
- the cost of preparation being generally low, since no specialized equipment is necessary;
- the possibility of incorporating both hydrophilic and lipophilic drugs (at the same time, if desirable), due to the presence of hydrophilic and lipophilic domains;
- the increased drug loading, since the amphiphilic interface can be viewed as an additional region for drug solubilization if compared to non-structured oily or aqueous vehicles;
- the penetration-enhancing ability.

From all of the abovementioned properties, the last is probably one of the most relevant when it comes to the use of microemulsions as a delivery system to the skin. This is, as previously mentioned, due to the need to overcome the well-known barrier function of the tissue to ensure an efficient delivery. 

## 4. Mechanisms of Penetration Enhancement

Several mechanisms have been proposed to explain the penetration-enhancing effect of microemulsions. Most likely, it is the overall combination of various mechanisms that result in the penetration-enhancing effect, and no mechanism in isolation seems to provide a sufficient explanation for the superiority of microemulsions compared to other systems. 

The first property relates to the small droplet size and large surface area/volume ratio. Several studies show an effect of formulation dispersed phase size on drug transport into/across the skin. Diazepam transport, for example, was enhanced by using formulations with a dispersed phase size below 0.5 micrometers (100–300 nm) compared to standard emulsions [28]. Significant improvement in transdermal delivery of betamethasone valerate and dipropionate, indomethacin, diclofenac, piroxicam and naproxen was demonstrated by Friedman *et al.* [29] using formulations with oil droplets of approximately 100 nm compared to standard creams. Not all systems with dispersed phase within the nanometer range are equally effective, though. As demonstrated by Shim *et al.* [30], lipid micelles increased the stratum corneum rigidity and decreased lidocaine hydrochloride delivery compared to liposomes with the same lipid components. The authors suggested that the aggregate morphology is relevant to its interaction with the stratum corneum. Taken together, these observations suggest that the reduced size of the internal phase of nanocarriers may favor penetration, but may not be sufficient, if isolated from other parameters, to promote it. It has also been suggested that microemulsion continuously fluctuating interfaces may increase drug mobility, aiding penetration [18].

The ability of microemulsions to increase the skin penetration of compounds can also be attributed to the action of individual constituents [31,32,33,34]. Certain surfactants monomers, components of the oil phase and other penetration enhancers incorporated in the system can diffuse to the skin surface and increase the permeation of drugs, either by disrupting the lipid structure of the stratum corneum (facilitating diffusion through the skin barrier) or by increasing the solubility of the drug in the skin (*i.e.*, increasing the partition coefficient of the drug between the skin and the vehicle) [15,27]. In a recent study designed to evaluate microemulsion component-induced changes in the stratum corneum, it was observed that all components perturbed this layer to a degree that was proportional to the level of the respective component present in the skin [35]. Oleic acid, for example, decreased the conformational order of lipids and induced phase separation [35]. The study also demonstrated that formulating components in a microemulsion increased their relative uptake into the stratum corneum compared to the pure components. It was suggested by the authors that the more efficient uptake from the microemulsion might imply the possibility of mutual enhancement. Based on these results, using a microemulsion instead of individual components may be advantageous.

Microemulsions have also been described to increase skin hydration. Since water is considered an enhancer [36], this effect may contribute to the formulation penetration-enhancing properties. In the study by Hathout *et al.*, the ratio of the peak amide I to amide II absorption increased (as determined using attenuated total reflectance Fourier transform infrared spectroscopy) as the percentage of water in the microemulsion increased, suggesting a gradual increase in the hydration of the stratum corneum [35]. Similar observations were reported by Gupta *et al.* [37]. Additionally, considering that several compounds used in the oil phase have occlusive properties (vegetable oils, for example) [38], it is possible that these components change the water gradient in upper skin layers by avoiding evaporation. Obviously, one must consider that different compounds exhibit different occlusive effects [39], and the properties of the oil phase may vary depending on the individual compounds included and their concentration. For example, the occlusive effects of oil-continuous emulsions with low water content seem to be more similar to the oil phase than that of W/O with high water content or O/W systems, which may exert an occlusive effect after unbound water evaporates [40].

Another property to which the penetration-enhancing effect of microemulsions has been attributed is the high drug loading capacity. The structural organization of the oil and aqueous phases and the presence of the surfactant-containing interface create additional solubility regions, increasing the loading capacity of microemulsions compared to non-structured vehicles [15,41]. The solubility of resveratrol, for example, was enhanced over 23-fold when it was incorporated in a self-microemulsifying delivery system compared to water [42]. The solubilities of progesterone and estradiol in microemulsion formulations containing Brij and miglyol were several orders of magnitude greater than those found in deionized water [43]. Even though the possibility of dissolving large amounts of lipophilic and hydrophilic drugs in microemulsions (without a concurrent increase in vehicle affinity) might increase the skin penetration of a molecule, due to larger concentration gradients, various studies have challenged this concept, demonstrating no clear relationship between increases in solubility and maximization of skin transport [44,45]. 

## 5. Microemulsion Composition, Characteristics and Skin Penetration

The ability of the final system to improve the transport of therapeutic agents into and across the skin is largely influenced by the internal structure and type of microemulsion used, as well as the composition and concentration of its components [27,46,47,48]. In fact, several studies demonstrate that the type of surfactant blend and/or co-surfactant used (as well as the ratio between them), the type of oil employed and the presence of penetration enhancers in microemulsions largely affect the skin penetration profile of a compound [27,46,47,48]. Depending on the physicochemical properties of the active substance, different types of microemulsions can be the optimal carrier. Thus, it is not enough to combine different components and obtain a microemulsion with suitable characteristics; it is necessary to find the appropriate composition and concentration of components to maximize its efficacy. 

Various surfactants, surfactant blends and co-surfactants have been used to obtain microemulsions for topical and transdermal delivery. A frequently used combination of surfactants is the mixture of caprylocaproyl polyoxylglycerides and polyglyceryl fatty acid esters. PEGylated fatty alcohols (e.g., Brij) are another important class of non-ionic surfactants. Aerosol OT (sodium bis(2-ethyl hexyl)sulfosuccinate), an ionic surfactant, offers the useful possibility of forming co-surfactant-free microemulsions [18]. In addition, polysorbates (Tween series) are often combined with other non-ionic surfactants to obtain microemulsions. Short chain alcohols are often employed as co-surfactants to aid microemulsion formation and increase the water solubilization capacity of the system [27].

Multiple compounds with varying characteristics have been used as components of the oil phase. Most of them are not conventional oils (neutral, nonpolar chemical substances), even though they generally display very low solubility in the aqueous phase, and many were selected because of their penetration-enhancing properties. Compounds that have been included as components of the oil phase range among fatty acids, alcohols, esters of fatty acids and alcohols, medium chain mono-, di- and triglycerides, terpenes, vegetable oils (such as jojoba oil) and other miscellaneous penetration enhancers. Among these, isopropyl myristate and oleic acid are probably the most frequently selected components of the oil phase [10,18,27]. Terpenes (such as menthol and limonene) have also been employed, due to their penetration-enhancing properties [49]. Table 2 summarizes examples of microemulsion composition and incorporated drugs used for a local effect and transdermal delivery. This table is far from giving a complete overview of the literature, but summarizes frequently used microemulsion components for topical/transdermal delivery over the past few years. The classification according to the main surfactant was based on Heuschkel *et al.* [18].

**Figure 1:**
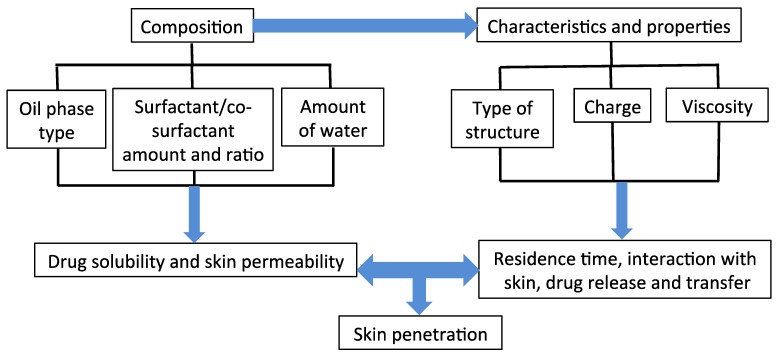
Microemulsion-related parameters influencing skin penetration and transdermal delivery of drugs and other active compounds. Depending on its physicochemical characteristics and system microstructure, a drug may be incorporated in the dispersed phase, dispersing medium and/or within the interface (at least partially). The term drug release was used here to address the process of a drug incorporated in any of these regions “leaving” the formulation, giving a measure of drug supply to the skin surface by the formulation. The term is generally used in the pharmaceutical literature. However, not every drug needs to be released, such as hydrophilic compounds dissolved in the dispersing aqueous phase. Hydrophobic drugs incorporated in non-polar domains must partition into stratum corneum, and some authors prefer the term “location exchange” for this process.

In the following subsections, the effect of microemulsion composition on its characteristics and, consequently, on its penetration-enhancing effect and potential of use as a topical delivery system will be discussed. Figure 1 summarizes the parameters that will be discussed.

**Table 2:** Examples of microemulsion composition and incorporated drugs used as transdermal delivery systems.

Surfactant blend or surfactant/co-surfactant	Components of the oil phase	Drug	Reference
*Aerosol OT-based microemulsions*			
Aerosol OT/butanol	isopropyl palmitate	hydrophilic and lipophilic anesthetics	[50]
Aerosol OT/Tween 85	isopropyl myristate	cyclosporine A	[51]
Aerosol OT	isopropyl myristate	5-fluorouracil	[37]
*PEGylated fatty alcohol (Brij)-based microemulsions*			
Polyoxyethylene (10) oleyl ether and1-hexanol	paraffin oil, isopropyl myristate or jojoba oil	sodium diclofenac	[52]
polyoxyethylene (20) cetyl ether and Span 80, ethanol, isopropyl alcohol, and propanol	soybean oil	sodium diclofenac	[48]
polyoxyethylene (10) dodecyl ether	tributyrin	progesterone	[53]
polyoxyethylene (10) oleyl ether, propylene glycol	glycerides of caprylic and capric acid	lycopene	[34]
polyoxyethylene (10) oleyl ether, propylene glycol, ethanol	monocaprylin	paclitaxel	[54]
*Polysorbate (Tween)-based microemulsions*			
Tween 80 and Span 20/ethanol	isopropyl myristate	sodium nonivamide acetate	[55]
Tween 80, ethanol, isopropanol or propylene glycol	eucalyptus oil	hydrocortisone	[27]
Tween 80/propylene glycol	oleic acid, menthol	triptolide	[32]
Tween 80/ethanol	isopropyl myristate, *n*-hexylamine and *iso*-octylamine	diclofenac	[56]
Tween 80 or Span 80/ ethanol or isopropanol	lecithin, oleic acid or isopropyl myristate	estradiol	[57]
Tween 80/propylene glycol, ethanol, isopropanol	limonene, 1,8-cineole, α-terpineol	curcumin	[49]
polysorbate 80/ medium-chain glyceride	isopropyl myristate	celecoxib	[58]
*PEGylated fatty acid esters-based microemulsions*			
PEG-8 glyceryl caprylate and caprate, diethylene glycol monoethyl ether (Transcutol P)	oleic acid	vinpocetine	[59]
PEG-8 caprylic and capric glycerides, polyglyceryl-6 dioleate	isopropyl myristate	diclofenac diethylamine	[60]
PEG-8 caprylic and capric glycerides/ polyglyceryl-6-dioleate	miglyol 812	ascorbyl palmitate	[61]
caprylocaproyl macrogol-8- glycerides, Transcutol P	oleic acid	terbinafine	[62]
polyoxyl-35-castor oil, ethanol	oleoyl macrogol-6 glycerides EP	aceclofenac	[63]
polyoxyl-35-castor oil, ethanol	oleic acid	penciclovir	[47]
*Phosphatidylcholine-based microemulsions*			
lecithin and linkers (sorbitan monooleate, sodium caprylate and caprylic acid)	isopropyl myristate	lidocaine	[64]
lecithin/*n*-propanol	isopropyl myristate	tetracaine hydrochloride	[65]
lecithin, isopropanol	isopropyl myristate, oleic acid	fluconazole	[66]
*Other systems*			
glyceryl oleate/polyoxyl 40 fatty acid derivatives/tetraglycol	isopropyl myristate	lidocaine	[67]
tocopheryl polyethylene glycol 1000 succinate	oleic acid, isopropyl myristate	temozolomide hexyl ester	[68]
decylglucoside, propylene glycol, phytosphingosine	medium chain mono-diglycerides	α-tocopherol and lipoic acid	[69]
decyl polyglucoside, lecithin, propylene glycol, 1,2-hexanediol	1-decanol, 1-dodecanol	miconazole nitrate	[70]
coco-glucoside, decylglucoside, lecithin	Labrafil M1944CS	5-fluorouracil	[71]

### 5.1. Influence of Microemulsion Components

#### 5.1.1. Components of the Oil Phase

Multiple compounds with varying characteristics have been used as components of the oil phase. Most of them are not conventional oils (neutral, nonpolar chemical substances), even though they generally display very low solubility in the aqueous phase, and many were selected because of their penetration-enhancing properties. In this section, we aim at comparing the influence of some of these compounds (and their mixtures) when used as the oil phase on the skin penetration mediated by microemulsions. Because many of the compounds used in the oil phase are penetration enhancers, distinguishing the colloidal effects of formulations from the molecular effect of the enhancers on drug transport is difficult and requires extensive comparative experiments, which most often have not been performed. Thus, for most studies, formulation and molecular effects cannot be dissociated. 

The choice of components of the oil phase may influence mainly three parameters: drug release, drug solubilization in the microemulsion (and thus, loading capacity) and skin permeability [72]. The study by Hashem *et al.* exemplifies the impact of oil phase components on drug release [73]. When lemon oil and isopropyl myristate were compared as the oil phase of microemulsions, the former provided a faster release and was more effective for enhancing the penetration of clotrimazole [73]. Even though oil phase components are most often selected to favor drug solubilization, previous studies suggest that changes in drug solubility do not necessarily translate into maximization of penetration and that the effects of oil phase components on the skin may be more relevant than increases in drug solubility. When designing microemulsions for ketoprofen delivery to the skin, Rhee *et al.*, compared triacetin, myvacet, oleic acid and isopropyl myristate as the oil phase [44]. Even though drug solubility was highest in triacetin (followed by myvacet, oleic acid and isopropyl myristate), oleic acid-containing microemulsions provided the highest permeation flux: ketoprofen flux was over six-fold higher than from myvacet oil-containing formulations (which dissolved approximately two-times more ketoprofen) [44]. The absence of a clear relationship between drug solubility in the oil phase and transdermal flux was also reported by Zhang *et al.* [45].

Specific characteristics of compounds used as the oil phase seem to be relevant for their penetration-enhancing effect. When the cyclic monoterpenes limonene, 1,8-cineole and α-terpineol, were compared as the oil phase of a microemulsion designed to increase curcumin delivery, the most pronounced effect was obtained with limonene [49]. The authors speculated about the relevance of the presence of non-polar or polar groups in the molecule, as well as the potentiation of the penetration-enhancing effect, due to the combination with ethanol used as the co-surfactant. Comparing microemulsions containing glycerides as components of the oil phase, it has been reported that increasing the number of acyl chains from mono- to triglycerides reduced the overall penetration-enhancing effect of the system, decreasing the delivery of the lipophilic lycopene [34]. This is most likely due to a stronger effect of the microemulsion containing mono- and diglycerides in increasing skin permeability, as measured by a reduction of skin electrical resistance. Decreasing the acyl chain length of unsaturated monoglycerides from 18 to eight carbons increased the penetration of lycopene, but not of ascorbic acid, in spite of the fact that the microemulsion containing the smaller monoglyceride decreased the skin electrical resistance (and thus, the barrier function) in a more pronounced manner [26]. This result suggests that the superiority of one oil phase mixture over the other also depends on the drug physicochemical properties. In fact, other studies support this observation. While Rhee *et al.* [44] reported the superiority of oleic acid to increase ketoprofen (lipophilic, log*P* > 2) transport, the opposite was observed by Chen *et al.*, when transdermal delivery of triptolide (hydrophilic, log*P* < 0) was studied [32]. The effect of the oil phase components may also depend on the microemulsion overall composition, as the effect of oleic acid has been reported to be more pronounced in more hydrophilic microemulsions [66]. 

#### 5.1.2. Surfactant/Co-Surfactant Blend

The fact that the type and concentration of surfactant, co-surfactant and the ratio between them affect microemulsion formation, size and shape of aggregates and water solubilization is well known [8,18,72,74,75]. Now, several studies have attempted to evaluate whether they also affect the penetration-enhancing ability of microemulsions. The lipophilicity and structure of the surfactant seems to play an important role in determining the microemulsion ability to release the active compound by affecting the packing of the interfacial layer [76]. Drug release modulation can affect transport into the skin, especially if the release is the limiting step for penetration [76]. Since surfactants and co-surfactants might potentially disrupt the stratum corneum, several authors postulated that increasing surfactant and/or co-surfactant content in microemulsions could aid drug transport into/across the skin [55,74]. Increasing surfactant concentration could also improve mass transport by increasing the number of “carriers” available for transport [77]. One might argue that using only surfactants (not in microemulsions) provides a similar effect. Considering that it has been recently demonstrated that formulating components in a microemulsion increased their relative uptake into the stratum corneum compared to the pure components, microemulsion use may be more advantageous [35].

A three-compartment (donor, skin, receiver) mass balance model was used by Yuan *et al.* to decouple partition and mass transport effects and better understand the effect of surfactant concentration on lecithin-linker microemulsion-mediated lidocaine penetration [77]. The model has three permeation parameters: the skin-donor partition coefficient, the donor-skin mass transfer coefficient and the skin-receiver mass transfer coefficient. Surfactant concentration displayed a relatively minor effect on the mass transfer coefficients, suggesting that permeation enhancement via skin disruption is not very relevant in the system investigated. Increasing surfactant concentration led to an increase in the concentration of lidocaine in the skin, which, in turn, produced a greater transdermal flux [77]. In O/W systems, the increase in lidocaine concentration in the skin was attributed to an increase in solubilization in the microemulsion, whereas in W/O systems, it was attributed to an increase in skin-donor partition. It should be acknowledged here that there are several more complex pharmacokinetic models developed to study skin transport and dermal absorption. Models like the one used by Yuan *et al.*, are relatively simple, in that the skin is represented as one compartment, which is in equilibrium with the vehicle and a receiving medium; others represent the skin as two compartments, with hydrophilic and lipophilic regions. These and other models have been reviewed in detail [78,79,80]. More complex models have also been developed, including the Anissimov model, in which the stratum corneum is represented by several compartments and transfer rate constants between them are linked to the diffusion time [81]. Models for drug transfer from the skin into receiving compartments have also been developed, considering that the blood capillaries present in the dermis play a role not only in solute removal from the dermis, but also in transport to deeper layers and underlying tissues [81]. To model drug distribution, Singh and Roberts have used a compartmental model assuming first-order diffusional mass transfer between the dermis and underlying tissue compartments with concurrent elimination due to blood flow [82]. 

However, when it comes to surfactant concentration, more is not always better, and one reason for this is the possible changes in the thermodynamic activity of the drug. Al Abood *et al.*, for example, observed that as the percentage of surfactant blend increased from 15% to 30% in microemulsions, the rate of ondansetron permeation increased [83]. However, further increases in the surfactant concentration reduced drug permeation, an effect that the authors attributed to a reduction on thermodynamic activity of the drug in the microemulsion when the surfactant concentration increased over a certain range. A general trend for decreasing the flux of sodium nonivamide acetate and prolonging the lag time with increasing the amount of Tween 80/Span 20 from 35% to 70% was observed by Huang *et al.* [55]. Increases in the ratio *surfactant:oil* have been described to increase the system microviscosity (even in more diluted systems) and result in the formation of a more densely and tightly packed structure [84,85]. As the microviscosity of the diffusion medium increases, the resistance for diffusion also increases [86]. 

In addition to the surfactant content, the relevance of the co-surfactant choice for the penetration-enhancing effect of microemulsions is supported by several studies. Since most often co-surfactants employed in microemulsions are short-length alcohols, their effect on skin permeability should not be underestimated. While assessing the flux of hydrocortisone across rabbit skin, El Maghraby [27] reported that ethanol produced the greatest effect, followed by propylene glycol and isopropanol. No correlation with drug release was observed, and the reported difference may result from a more pronounced effect of ethanol on the skin. Increasing the chain length of the co-surfactant from ethanol to isopropanol was recently demonstrated to decrease the flux of curcumin, while increasing the number of hydroxyl groups (as the co-surfactant changed from isopropanol to propylene glycol) increased the flux [49]. Among the compositions studied, the ethanol-containing microemulsion showed the most pronounced effect. 

The relevance of the ratio between surfactant and co-surfactant for penetration has also been reported. When the ratio between Chremophor EL and ethanol was close to 1:1 (*w*/*w*), the skin permeation of penciclovir from microemulsions was significantly increased, while its solubility in the microemulsion decreased [47]. Increasing drug solubility and affinity for the vehicle increased drug retention in the formulation [47].

### 5.2. Influence of Microemulsion Characteristics

Epithelial cells carry a negative charge upon their surface, due to the presence of negatively charged protein residues on the outer side of their membranes and of the ion pumps [87]. This notion has motivated the use of cationic formulations for topical and transdermal delivery, as stronger interactions with the skin and longer retention times of the system may lead to increases in drug penetration [70]. Our group has recently demonstrated that the addition of phytosphingosine and the generation of a positive charge in Brij-based microemulsions promoted a 1.5-fold increase in paclitaxel delivery into the skin layers, whereas the transdermal delivery was not significantly affected [54]. This effect was not related to increases in skin permeability, as demonstrated by the absence of changes on transepidermal water loss. A two-fold increase on α-tocopherol penetration into viable skin layers, but not on transdermal delivery, was achieved using cationic decylglucoside-based microemulsions [69]. Cationic decyl polyglucoside and lecithin-based microemulsions nearly doubled the miconazole nitrate accumulation in the skin, while transdermal transport was negligible [70]. These results confirm previous reports showing the benefit of a positive charge for increasing drug penetration, but also suggest the stronger influence on skin retention, at least for lipophilic drugs [88,89,90]. 

Compared to the charge, the effect of droplet size on microemulsion-mediated delivery is less studied. It has been generally accepted that reducing the droplet size from the macro- to nano-range increases penetration [28,91]. Diazepam transport, for example, was enhanced by decreasing the droplet size of formulations to below one micrometer (100–300 nm) [28]. Significant improvement in transdermal delivery of betamethasone valerate and dipropionate, indomethacin, diclofenac, piroxicam and naproxen was demonstrated by Friedman *et al.* [29] using formulations with oil droplets of approximately 100 nm compared to standard emulsions. On the other hand, the influence of this parameter comparing systems within the nanometer range is much less clear, and the formulations compared often present different compositions, making it difficult to estimate the effect of just the droplet size [6]. In a recent study, Sahle *et al.* observed that ceramides permeated into deeper layers to a higher extent from smaller droplet microemulsions, reaching even the receptor compartment (an index of transdermal delivery) [92]. However, the droplet size was not the only factor affecting ceramide delivery, with viscosity also playing a role. In a study conducted by Izquierdo *et al.*, no difference in the skin penetration of tetracaine was observed comparing formulations with various droplet sizes. Again, formulations also differed in composition [93]. 

Because microemulsions’ low viscosity is often considered a limitation for application to the skin, the effect of increasing viscosity by polymer addition on microemulsion delivery ability has been increasingly investigated. However, conclusions vary. It was suggested that increases in the residence time and promotion of stronger interaction between the formulation and skin could benefit skin penetration [53]. In agreement with this suggestion, the addition of silicon dioxide and a polymeric emulsifier (Pemulen TR1) increased the viscosity of microemulsions and improved progesterone permeation 1.24- and 1.63-fold, while decreasing the skin retention in relation to the unmodified microemulsion [53]. Similarly, carrageenan had a positive influence on sodium fluorescein permeation from microemulsions [94]. The opposite results were reported by Huang *et al.* [55]: the addition of 1% of polymers increased the viscosity of microemulsions, but decreased the flux and increased the lag time for the permeation of a derivative of capsaicin. The authors justify their results based on the fact that in very viscous formulations, drug diffusion and partitioning might be slower and/or occur at a smaller extent, limiting drug transport across the stratum corneum. Rozman *et al.*, reported that microemulsions thickened with carbomer delivered approximately the same amount of vitamins C and E in the epidermis compared to the non-thickened formulation, but the transdermal flux of vitamin C was approximately five-fold smaller [95]. Taken together, these studies suggest that changing formulation viscosity may have multiple outcomes. One reason for the disparity in results may be the fact that the thickened microemulsions have varying viscosity values (depending on the type and amount of thickening agent employed).

### 5.3. Influence of Water Content and Internal Structure

The content of water influences the microemulsion internal structure, which in turn, has been suggested to affect drug delivery to the skin. The skin penetration of lipophilic and hydrophilic drugs seems to be more pronounced with O/W than W/O systems. The transdermal flux of 5-aminolevulinic acid increased approximately 17-fold from O/W systems compared to bicontinuous systems, which in turn, was larger than flux from W/O microemulsions (which could not be quantified) despite the fact that the last displayed more surfactant/co-surfactant [96]. The authors observed that drug diffusion coefficient from the bicontinuous microemulsion was only 1.5-times smaller compared to the O/W system, and thus, other mechanisms, besides changes on drug mobility, may be important for the more pronounced effect of O/W systems. Microemulsions containing a higher percentage of the aqueous phase and O/W structure also improved sucrose delivery [97]. Lipophilic drugs that displayed more pronounced steady-state permeation flux and cumulative permeation as water content in microemulsions increased (and the microstructure was transformed from W/O to O/W) include ketoprofen and lidocaine [45]. For ketoprofen, enhancement ratio values of 1.86, 3.30 and 9.27 (compared to the control solution) for aqueous contents of 20%, 40% and 70% were observed, which corresponded to W/O, bicontinuous and O/W, respectively. Similar observations were reported for α-tocopherol; even though no transdermal delivery was detected, significantly more drug was detected in viable skin layers using an O/W compared to a W/O microemulsion [69]. 

Since microstructure transition in most of the studies was achieved by changing water content and, consequently, the ratio among components, the contribution of other factors to the skin penetration should not be underestimated. Increasing water as formulations transform from W/O to O/W may lead to: (i) a change in the thermodynamic activity of the drug (mainly lipophilic drugs) as a result of the changed drug solubility in the external phase, especially when the external phase content represents a higher proportion of the microemulsion than the other constituents; (ii) improved skin hydration; and (iii) changes in skin permeability, due to the varying of the concentration of the penetration enhancers often included in the surfactant blend or oil phase [10,11,45]. The influence of microemulsion aqueous content on stratum corneum hydration has been reported in several studies using attenuated-total reflectance Fourier transform infrared spectroscopy. Gupta *et al.* [37] observed that as the concentration of water increases in the microemulsion, the ratio of the amide I/II band gradually increases, suggesting an increase in the hydration of the stratum corneum. Similar observations were reported by Hathout *et al.* [35].

All these factors can mask the real effect of the internal structure. This was clear in one of our previous studies in which lycopene delivery to the skin from two microemulsions containing either a medium chain mono-diacylglyceride mixture or a triglyceride as the oil phase were compared [34]. The choice of oil influenced the internal structure of the system, as well as its effect on the skin barrier (as measured by changes on skin electrical resistance), with the microemulsion containing the mono-diacylglyceride mixture displaying a more pronounced effect. Thus, the influence of the internal structure (if any) in this study cannot be dissociated from the effects of the formulation components on the skin.

In an elegant study designed to isolate the influence of the internal structure on caffeine delivery to the skin as much as possible, Naoui *et al.* prepared microemulsions varying only on the ratio between surfactant and co-surfactant, while maintaining the ratio among surfactant, oil and water constant [98]. The O/W microemulsion increased caffeine permeation with respect to the bicontinuous, W/O microemulsion and control solution; however, the flux difference was much less pronounced than in previous studies, possibly because the effect of variations on the ratio among components and their total concentration was minimized. No difference in delivery to skin layers was observed among the studied formulations. With this study, the authors stressed the importance of considering the influence of the mass fraction of water, oil and surfactant blend.

## 6. Use of Microemulsions for Topical and Transdermal Delivery

As demonstrated in many studies discussed here, microemulsions are valuable formulations for the improvement of the localization of drugs within skin layers and for systemic delivery [8,9,15,18]. When *in vitro* penetration studies are conducted, delivery into the receptor compartment is often considered an index of transdermal delivery (in spite of the limitations of the method to mimic drug transfer to the blood) [81,99]. Obviously, a complete separation between cutaneous and transdermal delivery is not possible, but a careful formulation design allows some adjustment on the cutaneous/transdermal delivery balance to favor one over the other [74]. It is important to bear in mind that the formulation is not the only factor to influence this balance; drug characteristics also influence its fate and whether a more pronounced deep skin retention or absorption into the circulation will occur [100]. In the following sections, we will provide examples of microemulsion use to favor either cutaneous or transdermal delivery based on the site of action or treatment intended. This classification will be performed based on each study stated goal, even though the term transdermal has been used multiple times when a local effect at the skin is desired. 

### 6.1. Transdermal Delivery

As stated earlier in this manuscript, transdermal delivery suggests that a systemic effect is intended. A large number of studies focused on microemulsion use for the transdermal delivery of drugs for a wide variety of effects. In fact, a simple search on PubMed using the words microemulsion and transdermal provided 184 results (searched on 14 January 2014). Due to the large amount of material available, we will focus on studies from the last 10 years.

Due to the benefits of testosterone replacement in overtly hypogonadal men, a microemulsion-based transdermal delivery system for this compound was proposed [10]. The highest flux of testosterone was achieved (4.6 ± 0.6 μg cm^−2^ h^−1^) from a formulation containing 3% (*w*/*v*) of the active drug, 16% oleic acid, 32% Tween 20, 32% Transcutol^®^ and 20% water. Microemulsions composed of Cremophor, ethanol, oleic acid and water were used to improve the delivery of penciclovir [47]. The ratio between surfactant (Cremophor) and co-surfactant (ethanol) influenced the permeation-enhancing ability of the microemulsion: when this ratio was close to 1:1 (*w*/*w*), penciclovir permeation across the skin increased. 

A computerized statistical technique of response surface methodology with mixture design was used to optimize formulation composition and maximize transdermal delivery of citalopram [101]. The parameters investigated included a mixture of Brij 30/Brij 35 as the surfactant (at a ratio of 4:1, 20%–30%), isopropyl alcohol (20%–30%) and distilled water (40%–50%). Considering that the required drug transdermal flux to achieve an effect was about 1280 μg/h, *in vivo* studies showed that an optimized formulation containing 3% citalopram applied over an area of 3.46 cm^2^ could provide the minimum effective therapeutic concentration with no erythematous reaction. Other examples of compounds that had their transdermal delivery increased by the use of microemulsions include prodrugs of nicotinic acid [102], lidocaine [67], estradiol [57], tetramethylpyrazine [103], sodium diclofenac [48], buspirone hydrochloride [104], tolterodine tartrate [105], huperzine A [106], meloxicam [107], diclofenac epolamine [108], triptolide [32], vinpocetine [59] and olmesartan medoxomil [109].

Since the transfollicular pathway has been found to be important for percutaneous absorption of topically applied drugs, the ability of microemulsions to serve as a drug carrier for this route was recently studied [110]. Confocal laser scanning microscopy images suggested that hair follicles provided a pathway for the permeation of adapalene from microemulsions, and that the internal structure may play a role in transport, as drug penetration in the hair follicles increased from 0.109 ± 0.03 to 0.292 ± 0.094 μg as the microstructure shifted from oil-in-water to bicontinuous with an aqueous content increase. As discussed earlier in this manuscript, this shift was associated with composition changes, and thus, the real effect of the microstructure may be masked. The involvement of this route on microemulsion-mediated transdermal delivery was also observed by Hathout *et al.*, while investigating the transport of betahistine hydrochloride [111]. Confocal laser scanning microscopy images of the skin surface also revealed the involvement of the paracellular and intercellular routes, since high fluorescence was observed inside corneocytes and at the corneocyte border delineating its hexagonal structure.

### 6.2. Local Effect

There is a significant number of studies demonstrating that a more pronounced retention in the skin layers rather than percutaneous permeation can be obtained with microemulsions. This concept is largely used for formulations with cosmetic purposes. Decylglucoside-based microemulsions were proposed for topical delivery of ascorbic acid with the main purpose of using this compound not only as a protector from reactive oxygen species-induced skin damage, but also for skin lightening [112]. Microemulsions containing 20% dioctylciclohexane were more effective at increasing ascorbic acid localization in the epidermis at earlier time points, but formulations containing mineral oil seemed to provide superior protection of the compound against degradation. As in other studies discussed, this study supports the influence of microemulsion oil phase components on the skin penetration. The addition of phytosphingosine to confer a positive charge to sugar-based microemulsions was valuable in promoting the cutaneous retention of ascorbic acid and lycopene, while limiting transdermal delivery [69]. O/W microemulsions containing soy lecithin and decyl polyglucose were studied with the goal of obtaining waterproof, non-sticky and easily spreadable formulations for sunscreens. These formulations provided little permeation across lipophilic and hydrophilic membranes, indicating their potential as a sunscreen delivery system [113]. 

Even though the advantages of cutaneous localization of compounds for cosmetic purposes are more obvious, the benefits of this localization exist for other therapeutic groups, as well. Microemulsions were used to improve the localization of anesthetics into the skin and provided a faster onset of analgesia than commercial formulations [114,115]. Bicontinuous microemulsions composed of Aerosol-OT, Tween 80, isopropyl myristate and water were studied as topical delivery systems for cyclosporin A [51,116]. After *in vivo* topical application of the microemulsion, the deposition of the drug into skin and subcutaneous fat was almost 30- and 15-fold higher, respectively, than the concentrations obtained in the tissues after oral drug administration. Concentrations in the blood, liver and kidney were much lower following topical administration than that following oral administration, which suggests that adverse effects associated with systemic drug exposure could be minimized with this mode of application. Other compounds successfully delivered into the skin using microemulsions include lidocaine [67], alpha-tocopherol [117,118], temozolomide hexyl ester [68], ascorbyl palmitate [61], 8-methoxsalen [31], desmopressin acetate [119], paclitaxel [54], phthalocyanine tetrasulfonate [120] and linoleic acid [121].

### 6.3. Commercial Formulations and Microemulsions

A large number of patents considering mainly the topical use of microemulsions exist, and most of them focus on the cosmetic field. In fact, Avon, L’Oreal and Revlon (among many others) own patents on the use of microemulsions for skin, hair and nail care. It is not our goal to review patents, but in an attempt to show that this technology has been translated into products, we will provide a few examples of microemulsion-based commercially available formulations. The most well-known microemulsion-based formulation is Neoral^®^, a cyclosporine-containing peroral formulation. Several topical products claim to take advantage of microemulsions in their formulation, but information about composition and structure is difficult to find. Microemulsions of an amino-functional silicone polymer are available for use in shampoos, conditioners and stylizing gels (Dow Corning). Silicone microemulsions have also been employed in Kerastase hair care products. An anti-wrinkle microemulsion based on acetyl hexapeptide 3 (Auriga Int., Braine-l’Alleud, Belgium) is commercially available for skin care. A blend of PEG-6 caprylic/capric triglyceride, polyglycerol-6 dioleate, glyceryl caprylate/caprate was included in the system commercialized by Abitec, which, after water addition, forms microemulsions, whose suggested uses include sprayable lotions and as a vehicle for vitamin E acetate and sunscreens. 

## 7. Potential Adverse Effects of Microemulsions

A common concern related to microemulsion use for topical and transdermal delivery is their potential side effects, mainly the skin irritation potential and comedogenic effects. These are generally associated with exposure time, the composition and the concentration of components, like surfactants and the components of the oil phase. 

The comedogenic effect relates to the use of cosmetic formulations containing certain ingredients capable of producing comedones [122]. For a comedogenic effect, it has been generally accepted that a compound must penetrate into the follicle and produce hyperkeratosis [123]. Although there are no systematic studies evaluating the comedogenic properties of microemulsions, the effect of several compounds used in these formulations is known. For example, neat isopropyl myristate, isopropyl isostearate, decyl oleate, lauryl alcohol, lanolin and cocoa butter are considered comedogenic with varying intensity [123,124]. Ideally, only non-comedogenic components should be used to claim that a formulation is non-comedogenic, but considering that the effect is influenced by the concentration of a compound, the duration and the frequency of exposure, the properties of the final formulation may actually vary. In a comparative study, formulations containing one or more comedogenic compounds were considered non-comedogenic when their concentration decreased [122]. This provides one explanation for why water-continuous formulations are generally considered less comedogenic [123]. Additionally, the endpoint evaluated and model used may provide different results that further complicate formulation evaluation. Different results have been reported for compounds tested on the rabbit ear or human models and on assays employing macroscopic or histological evaluation or follicles and comedones [122].

The high concentration of surfactants often necessary for microemulsion formation has been a matter of concern when it comes to the formulation potential to cause irritation, and surfactants generally considered milder, such as those naturally available (like lecithin) and sugar-based (like polyglucosides), [66,89,125] have been employed to reduce the irritation potential. However, the inclusion of penetration enhancers as components of the oil phase may exacerbate the effect, especially because their irritation potential is generally perceived to increase as their penetration-enhancing effect becomes more pronounced [126]. The irritation potential of microemulsion surfactants and components of the oil phase generally relates to their ability to penetrate the skin, disrupt the stratum corneum, induce changes on keratinocytes and mediate the release of inflammatory cytokines [127]. 

In addition to animal and human-based assays, a large number of alternative methods with varying degrees of complexity are employed to evaluate the irritation potential of microemulsions, including *in vitro* cultures of fibroblasts and keratinocytes, red blood cell test, hen’s egg test chorioallantoic membrane and tridimensional bioengineered tissues [92,128,129,130]. Most often, the abovementioned assays involve comparisons of the microemulsion with negative controls (compounds that do not cause significant changes in viability, such as phosphate-buffered saline (PBS)) and positive controls (moderate or severe irritants, such as solutions of sodium lauryl sulfate at 0.5%–1%). However, the results reported are often difficult to compare as a consequence of the variety of assays, positive controls and exposure times employed. Regulatory organs have developed standardized score systems to guide results, but guidelines are not available for all assays [92,128,130]. 

In a general manner, microemulsions are considered less damaging than solutions of moderate and severe irritants (at 0.5%–1%). In the hen’s egg chorioallantoic membrane model assay, the irritation scores of the studied microemulsions (0.07–0.7) were close to that of saline and 12–100-fold smaller than that of sodium lauryl sulfate, considered a moderate-to-severe irritant [92]. According to the score system defined by ICCVAM, the formulation is a non-irritant (0–0.9: non-irritant; 1–4.9: slight irritation potential; 5–8.9: moderate irritation potential; and 9–21: severe irritation). Microemulsions have also been reported to cause a less marked reduction on cell and bioengineered cutaneous tissue viability after exposure for time periods varying within one and 24 h [74,131,132], but the viability seems to decrease as the ratio between water and surfactant + the oil phase decreases [54]. Our group and others have evaluated the irritation potential of microemulsions based on their time-dependent effects on human reconstructed tissue equivalents compared to negative (PBS) and positive (moderate irritants, such as Triton) controls [26]. Compared to PBS, the viability of the tissues treated with Triton (1%) was significantly reduced to 78.1% ± 9.5% after 2 h. An effect of similar magnitude was observed approximately after 5 h with the decylglucoside-based microemulsion, and the time necessary for this microemulsion to reduce tissue viability to 50% (ET_50_) was approximately three-times longer than that of Triton. So far, there has been no well-defined value of ET_50_ that ensures formulation safety, but several marketed topical formulations with good tolerability have ET_50_ values 3–9-times longer than Triton [133]. *In vivo* assays in rodents support these results. Polyglucoside-based microemulsions applied topically on rats for four days twice daily at 3-h intervals caused less marked erythema (a highest score of two, moderate erythema) than a solution of sodium lauryl sulfate (5%, a highest score of four, severe erythema) [71]. No visible signs of erythema (redness) or edema (swelling) were observed after topical treatment of shaved rabbits for 48 h with microemulsions containing Transcutol, Tween 80 and medium-chain mono-diglycerides [106]. 

## 8. Conclusions

When it comes to topical formulations in general, the choice of components and the ratio among them can dramatically change the system characteristics and delivery of active compounds to the skin. This is not different for microemulsions: the choice and amount of surfactant/co-surfactant, oil phase and water greatly affect the microemulsion, the solubilization capacity, the charge, the internal structure and the interactions with the drug. The resulting properties together with the ability of individual components to interfere with the cutaneous barrier are key determinants of skin penetration. When we consider all these parameters, it becomes easier to understand why there are so many published studies concerning microemulsions and skin, as well as why the results are often conflicting. Here, we hope to convey the message that even though microemulsions are considered relatively simple to obtain, they should not be viewed as an unsophisticated system, and a careful design is necessary if their potential as topical/transdermal delivery systems is to be maximized.
